# Microfluidic Devices for Analysis of Spatial Orientation Behaviors in Semi-Restrained *Caenorhabditis elegans*

**DOI:** 10.1371/journal.pone.0025710

**Published:** 2011-10-12

**Authors:** Kathryn E. McCormick, Bryn E. Gaertner, Matthew Sottile, Patrick C. Phillips, Shawn R. Lockery

**Affiliations:** 1 Institute of Neuroscience, University of Oregon, Eugene, Oregon, United States of America; 2 Institute of Ecology and Evolution, University of Oregon, Eugene, Oregon, United States of America; 3 Research and Development, Galois, Inc., Portland, Oregon, United States of America; Harvard University, United States of America

## Abstract

This article describes the fabrication and use of microfluidic devices for investigating spatial orientation behaviors in nematode worms (*Caenorhabditis elegans*). Until now, spatial orientation has been studied in freely moving nematodes in which the frequency and nature of encounters with the gradient are uncontrolled experimental variables. In the new devices, the nematode is held in place by a restraint that aligns the longitudinal axis of the body with the border between two laminar fluid streams, leaving the animal's head and tail free to move. The content of the fluid streams can be manipulated to deliver step gradients in space or time. We demonstrate the utility of the device by identifying previously uncharacterized aspects of the behavioral mechanisms underlying chemotaxis, osmotic avoidance, and thermotaxis in this organism. The new devices are readily adaptable to behavioral and imaging studies involving fluid borne stimuli in a wide range of sensory modalities.

## Introduction

The ability to migrate up or down chemical and thermal gradients is a key component of spatial orientation behaviors from single-cell microorganisms [1] to humans [2]. Such abilities – called chemotaxis and thermotaxis, respectively – are essential to biological processes as diverse as reproductive fertilization, development, the immune response, feeding, and habitat selection. Analysis of the behavioral mechanisms of chemotaxis and thermotaxis can be simplified by the use of step-like spatial or temporal gradients. Step gradients are advantageous because orientation responses in many organisms are triggered by detection of changes in sensory input rather than its absolute magnitude. Therefore, in the case of a step gradient, the effective stimulus is confined to a small region of space or time, making it easier to establish causal connections between stimuli and responses.

The formation of spatial and temporal step gradients can be challenging in the fluid environments in which many orientation behaviors are investigated. Spatial step gradients are problematic because they are rapidly degraded by antagonistic processes such as convection and diffusion, whereas temporal step gradients are problematic because of long switching times when perfusing macroscopic experimental chambers. At microfluidic scales, however, spatial step gradients are readily formed by combining laminar streams of distinct fluids [3]. This feature has led to the development of microfluidic step gradient generators for the study of spatial orientation behaviors in several types of widely used microorganisms including bacteria [4], *paramecium* [5], sperm cells [6], and nematodes [7], [8]. Temporal steps are also comparatively easy to generate in microfluidic devices because low fluid volumes lead to reduced switching times [7], [8].

In the current generation of microfluidic devices for studies of spatial orientation, organisms move freely within the device. Although beneficial in many respects, freedom of movement has two key limitations. First, freely moving microorganisms can be difficult to track, making longitudinal studies impractical. Second, in the case of spatial gradients, when, how often, and at what angle a particular individual encounters the step are uncontrolled variables that depend entirely on the organism's behavior. Analysis of orientation mechanisms would therefore be accelerated by a general method for presenting step gradients to semi-restrained individuals.

As a first step toward addressing this need, we sought to develop step-gradient devices for studying orientation behaviors in the nematode worm *Caenorhabditis elegans*. *C. elegans* is an unusually well-described organism that is widely used as a model system in biological and biomedical research [9]. Adults of this species are 1 mm long and ∼80 um wide, making them large enough to be manipulated easily, yet small enough to be compatible with microfluidic devices [10]. In addition, *C. elegans* exhibits a diverse repertoire of spatial orientation behaviors including chemotaxis to gradients of soluble compounds and odorants [11] thermotaxis to preferred temperatures [12], and avoidance of regions of high osmolarity [13]. Two main behavioral strategies have been proposed to explain spatial orientation behaviors in *C. elegans*. The first is a biased random walk, also known as klinokinesis, in which the frequency of large turns is modulated by the rate of change of attractant concentration [14]. The second is a directed strategy known as klinotaxis, in which the animal's course is continuously corrected toward the line of steepest ascent up the gradient [15]. A necessary condition of klinotaxis is that course corrections occur as a result of alternating lateral displacements of the pertinent sensory organs [16]. Neuronal analysis of these behaviors reveals universally applicable circuit motifs for behaviors in higher organisms [17].

Here we describe a pair of microfluidic devices that reliably deliver step gradients in chemical concentration and temperature to semi-restrained *C. elegans*. The nematode is clamped at its midsection by a vacuum-assisted restraint aligned with the border between two laminar fluid streams. The head and anterior body, which are free to move, exhibit the side-to-side head swings characteristic of normal locomotory undulation in nematodes. The devices are capable of delivering both spatial and temporal steps. To demonstrate the utility of these devices, we investigated the behavioral mechanisms of orientation to chemical, thermal, and osmotic gradients at the resolution of individual head swings in *C. elegans* for the first time. Using spatial steps, we found that locomotion is biased toward favored chemical and thermal conditions during individual head swings, supporting the hypothesis that *C. elegans* employs klinotaxis in both modalities. Using temporal steps, we found that worms transiently increase the probability of initiating avoidance responses following sudden changes in osmolarity, indicating that *C. elegans* may employ an unusual klinokinesis strategy to orient to osmotic cues. The device is readily adaptable to a wide range of studies including classical conditioning and neuronal imaging in intact, behaving animals.

## Results

### Design of the chemosensory device

**Figure 1:**
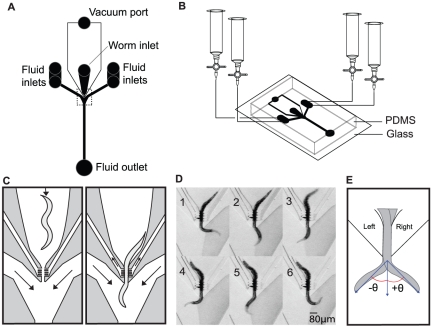
Design of the chemosensory device. (A) Top view showing the layout of channels, ports, inlets, and outlets. The region enclosed by the dashed rectangle is expanded in (C) and (D). (B) Perspective view showing the arrangement of fluid reservoirs and valves for switching fluids. (C) Schematic of the worm loading process. Arrows represent direction of flow in fluid and vacuum channels. Left panel: The worm is pushed toward the restraint via a pressurized syringe (not shown) attached to worm inlet. Right panel: Once the worm is in place, an external vacuum source is applied and the syringe is de-pressurized. (D) Composite image with one stream dyed to demonstrate laminar flow during a complete head swing cycle. (E) Definition of head angle *θ* used for quantification of behavior. Undulations occur in the dorso-ventral plane. The terms “left” and “right” refer to the arms of the device.

The chemosensory device consists of a Y-shaped channel formed in a single layer of PDMS bonded to a glass substrate (Fig. 1A,B). Solutions containing chemosensory stimulants enter via inlets located at the ends of each arm and are removed via an outlet at the base of the stem. The solution flowing in each arm is selected manually by means of stopcocks attached to the fluid reservoirs, or automatically by a bank of solenoid valves (not shown); flow rate is regulated by a peristaltic pump attached to the outlet. The worm is positioned at the center of the Y where the fluids from the arms converge. We found that a simple passive clamp in the form of a channel with a narrow constriction was insufficient to prevent the worm from escaping from the device. This problem was solved by applying a vacuum to both sides of the worm via a manifold of 10 um wide ports (Fig. 1C). The restraint immobilizes the middle third of the worm leaving the anterior (head) and posterior (tail) portions free to move. At the start of an experiment, the worm is captured in a fluid filled tube, inserted into the worm inlet, and positioned in the restraint; the vacuum is activated after the worm is in position.

The central feature of the design is that the two streams meet without mixing at the point of confluence (Fig. 1D). Thus, when the streams contain different concentrations of a chemical stimulus, a step-like chemical gradient is formed across the region in which the worm's anterior portion is free to move. Alternatively, the device can be used to deliver temporal concentration steps. This is done by flowing the same solution through both arms then simultaneously switching them to a different solution. We refer to these methods as the spatial and temporal modes of operation, respectively.

### Performance

#### Animal behavior

On an agar substrate, a freely crawling worm lies on its right or left side such that undulations occur in the dorso-ventral plane. We found that when inserted into the device, the worm invariably adopts such an orientation. We observed that the anterior portion of the animal moved in a coordinated manner that qualitatively resembled the locomotion of unrestrained worms (Fig. 1D). Unrestrained worms exhibit two distinct modes of locomotion: crawling and swimming, which have undulation frequencies of ∼0.8 Hz and ∼2.1 Hz respectively [18]. Undulation frequency in the device was 0.34±0.11 Hz (*n* = 17 worms, s.e.m.) suggesting that the worms were crawling rather than swimming.

#### Temporal precision of stimulus delivery

To assess the chip's performance when being operated in temporal mode, we measured the time course of solution exchange. This was done by switching the flow in one of the arms from a clear solution to one containing an opaque substance (food dye) and measuring the time course of average luminance changes in a region of interest located near the convergence of the two streams. Solution exchange was complete in 0.53±0.19 sec (*n* = 5 trials, s.e.m.), which is much shorter than the period of undulation in the device (2.9 sec).

### Behavioral responses to step gradients in chemoattractant concentration

**Figure 2:**
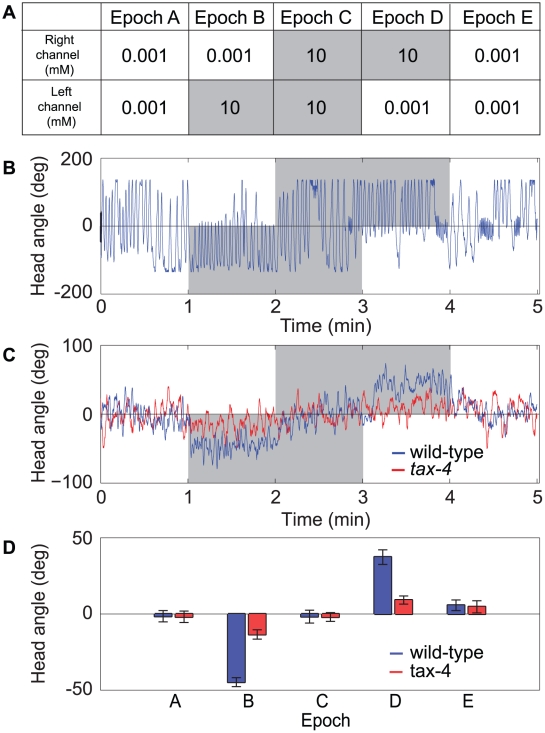
Behavioral responses to step gradients in chemoattractant concentration. (A) Stimulus protocol for chemosensory experiments. Values are the concentration of NaCl in mM. All epochs are one minute in length. Shading indicates timing and location of high concentration fluid. (b) Head angle versus time for a wild-type animal in response to the stimulus protocol in (A). (C) Ensemble averages of head angle for wild type animals and *tax-4* mutants. (D) Mean head angle of wild type animals and *tax-4* mutants by stimulus epoch for data shown in (C). Error bars are s.e.m.

To demonstrate the new types of data that can be acquired by the chemosensory device, we first examined the behavioral mechanism of directed orientation responses in *C. elegans*. These responses are hypothesized to involve klinotaxis, i.e., modulations of head angle based on concentration changes sensed during the lateral component of head movements associated with individual undulations [15]. However, this hypothesis is based on analysis of the trajectory of the nematode's centroid rather than detailed analysis of alterations in head swings that may be an essential component of the orientation mechanism. To test the klinotaxis hypothesis at the resolution of individual head swings, we used the device in the spatial operating mode. This was done by subjecting individual worms to an alternating series of symmetrical and asymmetrical patterns of the attractant concentration (NaCl), in five contiguous epochs (Fig. 2A). Symmetrical stimulus epochs (either 10 mM or 0.001 mM NaCl) were interleaved with asymmetrical stimulus epochs (10 mM versus 0.001 mM NaCl) in a counterbalanced design. Behavior was quantified in terms of angular displacement *θ* of the tip of the worm's head as shown in Fig. 1E. Dorsoventral undulations continued throughout the experiment (Fig. 2B). Data from individual worms showed that head swings from the high to the low concentration side in asymmetrical epochs were almost always truncated at the interface between the two solutions (*θ* = 0), whereas this truncation was absent during symmetrical epochs, except immediately after switching to symmetrical solutions. Group data, in the form of ensemble averages of *θ*, showed that undulations were strongly biased toward the high concentration side (Fig. 2C,D; ANOVA with post hoc contrast, *p*<0.001, *n* = 17). Average *θ* during symmetrical epochs was not significantly different from zero (post hoc contrast, *p*>0.05, *n* = 17), indicating that the bias seen in asymmetrical epochs was specifically the result of the concentration differences presented. We conclude that *C. elegans* is capable of modulating head swings in response to concentration changes sensed during the lateral component of head movements, at least in the case of concentration changes on the order of 10 mM. This finding provides direct confirmation of the klinotaxis hypothesis.

Behavioral analysis of chemotaxis mutants is a powerful means of investigating the cellular and molecular basis of chemotaxis [11]. To demonstrate the ability of the chip to identify novel quantitative phenotypes in chemotaxis mutants, we examined the behavior of the mutant *tax-4*, which has a well-characterized defect in chemotaxis [19]. This defect has been traced to impaired function of the TAX-4 protein, which is an ion channel required for chemosensory transduction in *C. elegans* [20]. We found that mean head angle during asymmetrical epochs was significantly reduced in the mutants relative to wild type controls (Fig. 2C,D; *t*-test, *p*<0.001, *n* = 18). This result demonstrates that *tax-4* is required not only for chemotaxis in general, but also for klinotaxis in particular, thereby providing a new behavioral phenotype for this mutation.

### Behavioral responses to temporal steps in osmolarity

*C. elegans* avoids a droplet of hyperosmotic fluid placed in its path by initiating a bout of reverse locomotion [13], [21]. The main chemosensory neuron for detecting changes in osmolarity in *C. elegans* is ASH. This neuron is activated not only by sudden increases in osmolarity (on responses), but also by sudden decreases in osmolarity (off responses) [22]. Whether osmotic avoidance is initiated by on responses, off responses, or both is unknown. This is because a freely moving worm immediately withdraws from an osmotic stimulus, thereby superimposing on and off responses.

**Figure 3:**
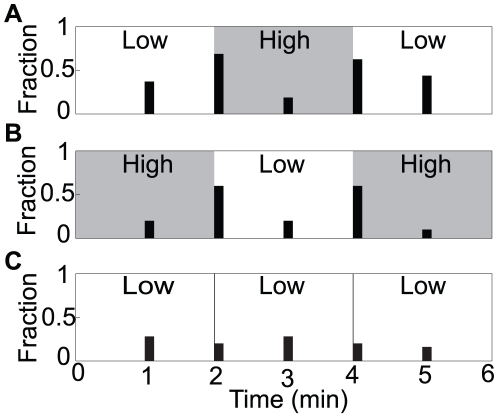
Behavioral responses to temporal steps in osmolarity. (A–C) Data are quantified as the fraction of worms that initiated at least one reversal in the 5 second interval beginning at the times shown on the abscissa. Shading indicates high osmolarity. Numbers of replications in A–C were 16, 10, and 25, respectively.

To address this issue we used the device in its temporal operating mode. This was done by presenting two groups of worms with a temporal series of spatially symmetrical stimulus epochs. The series alternated between solutions of low and high osmotic strength (370 mOsm and 1000 mOsm, respectively) according to the counterbalanced design shown in Fig. 3A,B. As a control for possible mechanical artifacts of solution switching, a third group of animals was presented with mock solution changes between two reservoirs containing low osmolarity fluid (Fig. 3C). Behavior was quantified in terms of the fraction of worms that initiated at least one reversal in a 5 second interval at the beginning and the middle of each epoch. The switch from low to high osmotic strength caused an increase in reversal probability relative to controls (Fisher's exact test, *p*<0.0001, *n* = 51), as did the switch from high to low osmotic strength (Fisher's exact test, *p*<0.001, *n* = 51). Reversal probability was unaffected in the control group, indicating that reversal bouts were specifically the result of changes in osmolarity (Fisher's exact test, *p*>0.05, *n* = 25). These results suggest that both on responses and off responses in ASH are sufficient to generate avoidance responses.

### Design of the thermosensory device

**Figure 4:**
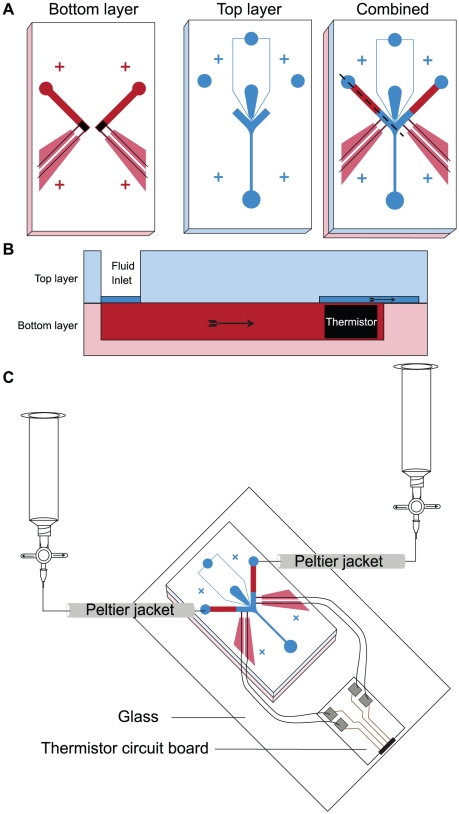
Design of the two-layer thermosensory device. (A) Top view showing the layout of channels in each layer before and after assembly (combined). The bottom layer (red) contains the arms of the device with embedded thermistors and wires (black). The top layer (blue) contains the worm restraint and the point of convergence of the fluid streams. (B) Side view through the plane indicated by dotted line in the combined view in (A). Saturated colors represent the channels through which fluid flows whereas less saturated colors represent bulk PDMS. Arrows indicate direction of flow. (C) Perspective view showing the arrangement of fluid reservoirs, Peltier tubing jackets for heating and cooling, and electrical contacts. Peltier jackets are controlled by an external temperature control module (not shown).

The thermosensory device is designed to present the worm with a step-like thermal gradient. It is similar to the chemosensory device except that the arms of the Y-shaped channel are enlarged to accommodate a pair of thermistors located immediately upstream of the point of confluence. The thermistors are used to monitor the temperature of the two streams. This modification necessitated a two-layer design in which upper and lower PDMS layers are bonded with their feature sides apposed (Fig. 4A). The upper layer contains the worm inlet, vacuum port and manifold, and the stem of the Y-shaped channel, including the point of confluence. The lower layer contains the arms of the Y-shaped channel with embedded thermistors and side channels for thermistor wires. Prior to assembly, the thermistors are fixed in place by filling the side channels with optical adhesive which also seals these channels against fluid leaks. Fluid flowing in the lower layer reaches the upper layer by traveling up and over the thermistors (Fig. 4B). At the flow rates used in these experiments, we estimate that the travel time between thermistor and worm was less than 500 milliseconds. The temperature of the fluids entering each arm of the Y-shaped channel was manually regulated by separate Peltier devices (Fig. 4C). The thermosensory device was operated only in spatial mode because of the length of time (30 seconds) required for temperature in the arms to equilibrate. After equilibration, temperature was stable to ±0.5°C for the duration of a typical experiment (∼5 min).

### Behavioral response to step gradients in temperature

**Figure 5:**
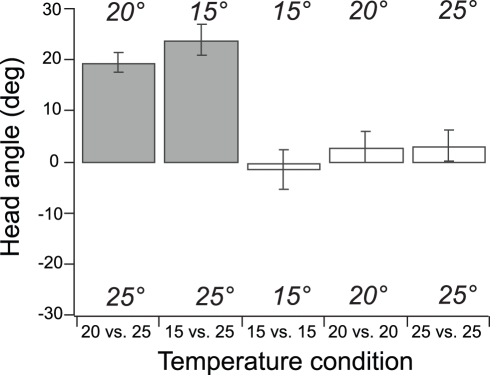
Behavioral response to step gradients in temperature. Mean head angle was computed as in Fig. 2D. Responses to five different temperature conditions are shown. Worms were cultivated at 20°C. Shaded bars indicate asymmetrical temperatures. In each condition, the direction of the bar shows preferred temperature, if any. Error bars are s.e.m.

To demonstrate the type of insights that can be gained with the thermosensory device, we investigated the behavioral mechanisms of thermotaxis. Thermotaxis in *C. elegans* involves klinokinesis [23], [24], but whether klinotaxis also plays a role is unknown. To address this question, we cultivated worms under conditions known to make them prefer a temperature of 20°C [25] and exposed them in the device to fluid streams of 20 and 25°C. Behavior was quantified in terms of average head angle (Fig. 1E) over a three minute trial such that positive angles corresponded to the head visiting the cooler stream. Temperature was randomized with respect to the left and right sides of the chip and the dorsoventral orientation of the worm. Under these conditions, head angle was clearly biased toward the 20°C stream (Fig. 5; *t*-test, *p*<0.01, *n* = 16). No such bias was observed in worms exposed to streams of the same temperature (*t*-test, *p*>0.05, *n* = 19 for the 15, 20, and 25°C conditions), indicating that the effect was specific to the temperature difference. These findings are consistent with a role for klinotaxis during thermal migration in *C. elegans*, at least in steep thermal gradients. In a second experiment, worms cultivated at 20°C but exposed to streams of 15°C and 25°C were biased toward the former (*t*-test, *p*<0.01, *n* = 19). Thus, in a forced choice between two temperatures equidistant from the preferred temperature, worms appear to prefer the cooler temperature, consistent with the finding that in *C. elegans*, thermotaxis down a gradient is more robust than thermotaxis up a gradient [26], [27], [28].

## Discussion

The device described here makes it possible to deliver spatial and temporal step gradients to semi-restrained *C. elegans*. A key feature of the device is a vacuum-assisted restraint that aligns the long axis of the worm with the stable border between two solutions. As a result, the response of individual worms to multiple border crossings can be examined. Additionally, the restraint ensures that the worm always encounters the border at the same angle. Together, these two aspects of the device improve the statistical power of step response experiments relative to those that rely on incidental border crossings in freely moving worms [8], [29].

We used the device to investigate the spatial orientation strategies in *C. elegans* at the resolution of single head swings. By presenting spatial concentration steps of a chemoattractant, we were able to confirm the hypothesis that *C. elegans* can perform klinotaxis, at least in response to large concentration changes. However, our findings do not exclude the possibility that a different behavioral mechanism is utilized when concentration changes are small, nor do they necessarily imply that the same neuronal mechanism is involved. In analogous experiments using a thermal step, we found that *C. elegans* is also capable of using klinotaxis when navigating steep thermal gradients. Both types of experiment would have been difficult or perhaps impossible to interpret in the case of freely moving worms in continuous gradients where concentration, or temperature, changes throughout the head swing.

We also used the device to investigate responses to temporal steps in osmolarity. In these experiments we found that sudden changes in osmolarity trigger reversals not only when osmolarity rises but also when it falls. We hypothesize that the latter type of response was missed in previous studies of osmolarity responses because the unrestrained worm withdraws so quickly from the stimulus that on and off responses are superimposed. This finding highlights the value of precise temporal control of stimulus presentation.

The device is readily adaptable to many other types of experiments. Orientation responses of wild type and mutant worms can now be studied at high resolution in response to spatial and temporal step gradients of a wide range of fluid borne cues including pH [30], odorants [31], pheromones [32], oxygen [33], carbon dioxide [34], and environmental toxins [35]. Using the device in temporal mode, it should be possible to associate sensory cues with appetitive or aversive stimuli in simple classical conditioning experiments [36]. The effects of such treatments could then be tested by presenting the conditioned cues against each other in the device's spatial mode. With minor modifications, the device could be combined with on line image processing to deliver spatial or temporal steps that are time-locked to particular behaviors or postures of the animal. Such experiments would make it possible to test dynamical models of sensorimotor integration in *C. elegans* [37]. Finally, the transparency of the device makes it compatible with optogenetic approaches in which genetically targeted probes are used to record [38] and stimulate [39], [40] individual neurons while the animal is moving. Such experiments are likely accelerate our understanding of the neuronal basis of behavior in this key model organism.

## Materials and Methods

### Device Fabrication

We fabricated both devices using standard soft lithographic methods [41]. Full resolution electron files of photomasks are available in Supplementary Information (Figures S1, S2, and S3). A silicon wafer master for the chemosensory device was created by exposing a 60 µm layer of SU-8 2025 resist (Microchem, Newton, MA) through a transparency mask and dissolving away unexposed material. Masters for the top and bottom layers of the thermosensory device were created by exposing 60 and 500 µm layers of SU-8 2025 and SU-8 2050, respectively. Masters were treated with chlorotrimethylsilane (Aldrich, St. Louis, MO) to prevent adhesion of PDMS to the master. Masters were replica molded in PDMS (Dow Corning Sylgard 184, Corning, NY). Holes for ports and inlets (1.0 or 1.5 mm diam.) were formed using a biopsy punch. Thermistors (Panasonic NTC JZ(0201) and associated wires were embedded into channels in the lower layer of the thermosensory device with UV-sensitive optical glue (Norland 81, Norland, Cranbury, NJ). PDMS castings were bonded to their respective substrates after 30 sec exposure to an oxidizing air plasma. To facilitate alignment in two layer devices, castings were moistened with methanol immediately prior to assembly. As the methanol evaporated, the feature sides came into contact and bonded tightly. The assembled device was then placed in a polycarbonate clamp and thermistor wires were soldered to an external circuit board.

### *C. elegans* cultivation

Synchronous populations of wild type (N2) and mutant (*tax-4(p678*)) *C. elegans* were grown on standard nematode growth medium (NGM) plates seeded with *E. coli* OP50 as described [42]. On the first day of adulthood, worms were picked to an unseeded NGM plate and allowed to crawl free of any co-transported bacterial food. Worms used in chemosensory and osmosensory experiments were starved for 30–60 minutes prior to experiments whereas worms used in thermosensory experiments were maintained in food until use.

### Solutions

In chemosensory experiments, the solutions contained (in mM) 1 CaCl_2_, 1 MgSO_4_, 10 HEPES, and NaCl as indicated in the text. Glycerol was added to achieve a total osmolarity of 370 mOsm. Similar solutions were used in osmolarity experiments except that the concentration of NaCl was 1 µM and osmolarity was adjusted to 370 mOsm or 1000 mOsm in the low and high osmolarity solutions, respectively. In thermosensory experiments, the solution used was S Basal [42]. All solutions were filtered with a pore size of 0.22 µm prior to use. In thermosensory experiments, temperature was regulated by a peltier temperature control system (TC2BIP with CH module, Cell MicroControls, Norfolk, VA). Flow rates through the device were 10–15 mL per hour.

### Data collection and analysis

Video recordings of worm behavior (30 frames/sec) were analyzed in MATLAB using a custom routine to compute head angle *θ* in each image. Briefly, frames were first masked and thresholded to obtain an image of the worm. The centerline of the worm was then obtained by a skeletonization procedure. Starting at the position of the restraint, the centerline was traversed to find the tip of the head, defined as the point furthest from the restraint. Initiation of reversals was scored manually by an observer who was blind to experimental condition. Reversal behavior was defined as propagation of the undulatory wave in the posterior to anterior direction as previously described [43].

## Supporting Information

Figure S1 **Photomask design for the chemosensory device.**
(PDF)

Figure S2 **Photomask design for the thermosemsory device, upper layer.**
(PDF)

Figure S3 **Photomask design for the thermosensory device, lower layer.**
(PDF)
