# Supplementary figures and images for: Microfluidic Devices for Analysis of Spatial Orientation Behaviors in Semi-Restrained Caenorhabditis elegans
Source: PLoS One. 2011 Oct 12;6(10):e25710. doi: 10.1371/journal.pone.0025710 (PMC3192130; doi:10.1371/journal.pone.0025710)

# River-7 06Oct08

1 m

North 00 m

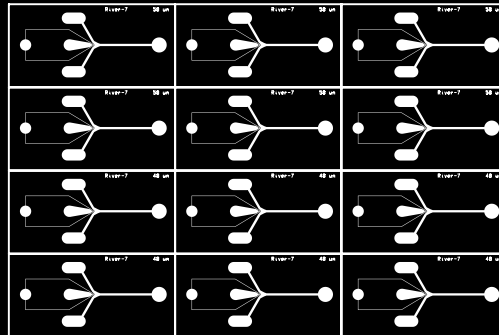

Supplement: Figure S1 — Photomask design for the chemosensory device. (PDF) [file pone.0025710.s001.pdf]

HotCold-1  
18 Dec 08  
60  $\mu\text{m}$  / 300  $\mu\text{m}$  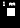

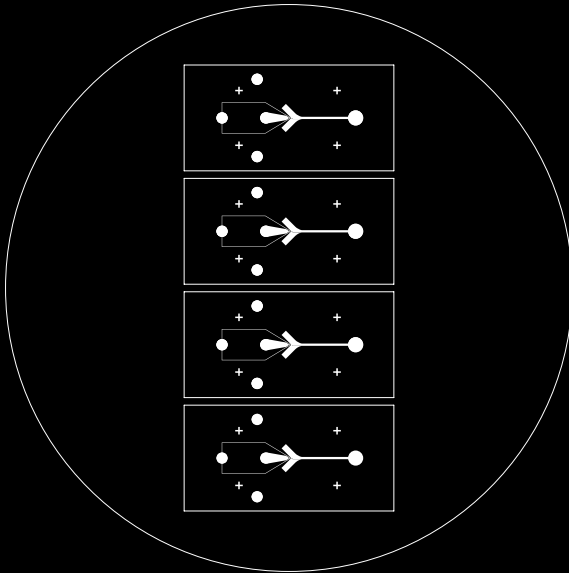

Supplement: Figure S2 — Photomask design for the thermosemsory device, upper layer. (PDF) [file pone.0025710.s002.pdf]

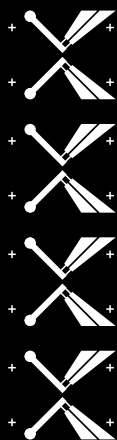

Supplement: Figure S3 — Photomask design for the thermosensory device, lower layer. (PDF) [file pone.0025710.s003.pdf]
